# Thymol‐Rich *Lippia thymoides* Mart. & Schauer (Verbenaceae) Essential Oil: Seasonal Chemical Variation, Antioxidant Capacity, *Artemia salina* Toxicity, and AChE Docking

**DOI:** 10.1002/cbdv.71715

**Published:** 2026-09-20

**Authors:** Jorddy Neves Cruz, Sebastião Gomes Silva, Angelo Antonio Barbosa Morais, Anderson Bentes de Lima, Antonio Rafael Quadros Gomes, Moises Hamoy, Eloisa Helena de Aguiar Andrade

**Affiliations:** ^1^ Adolpho Ducke Laboratory, Botany Coordination Emílio Goeldi Museum Belém Pará Brazil; ^2^ Laboratory of Experimental Pharmacology State University of Pará Belém Pará Brazil; ^3^ Laboratory of Pharmacology and Toxicology of Natural Products, Biological Sciences Institute Federal University of Para Belém Pará Brazil; ^4^ Postgraduate Program in Biodiversity and Biotechnology (BIONORTE) State University of Pará Belém Pará Brazil

**Keywords:** acetylcholinesterase, brine shrimp lethality assay, molecular docking, radical scavenging assays, thymol

## Abstract

Essential oils are chemically complex natural products whose biological profiles may vary with volatile composition. This study integrated chemical characterization, antioxidant evaluation, *Artemia salina* lethality testing, and acetylcholinesterase (AChE) docking to investigate thymol‐rich *Lippia thymoides* Mart. & Schauer (Verbenaceae) essential oil (LTEO) from leaves collected in September 2016 and February 2017. LTEO yield was higher in February 2017 (2.70%) than in September 2016 (2.24%). Overall, 38 constituents were identified, with thymol as the major compound in both samples (32.13% and 30.41%, respectively). The February sample showed higher proportions of monoterpene hydrocarbons, mainly p‐cymene, γ‐terpinene, and myrcene, whereas the September sample contained higher proportions of oxygenated monoterpenes, thymol acetate, and (E)‐caryophyllene. Both samples showed radical‐scavenging capacity, with numerically higher Trolox equivalent antioxidant capacity (TEAC) values for September. LTEO induced concentration‐dependent lethality in *A. salina*, with LC_50_ values of 71.08 ± 7.06 µg mL^−^^1^ for September and 60.06 ± 6.12 µg mL^−^^1^ for February. Docking indicated that (E)‐caryophyllene and thymol acetate had the most favorable dimensionless MolDock Scores. These findings reveal collection‐period‐related chemical differences and preliminary antioxidant, toxicological, and predictive AChE‐interaction profiles. Interactions were dominated by hydrophobic and van der Waals contacts, supporting their interpretation as preliminary molecular evidence requiring future biochemical validation studies.

## Graphical abstract

Thymol‐rich *Lippia thymoides* essential oil shows collection‐period‐related chemical shifts that align with changes in radical‐scavenging capacity, *Artemia salina* lethality, and predicted AChE interactions. This integrated chemical, biological, and computational profile highlights the importance of collection timing in interpreting the preliminary antioxidant and toxicological potential of essential oils.

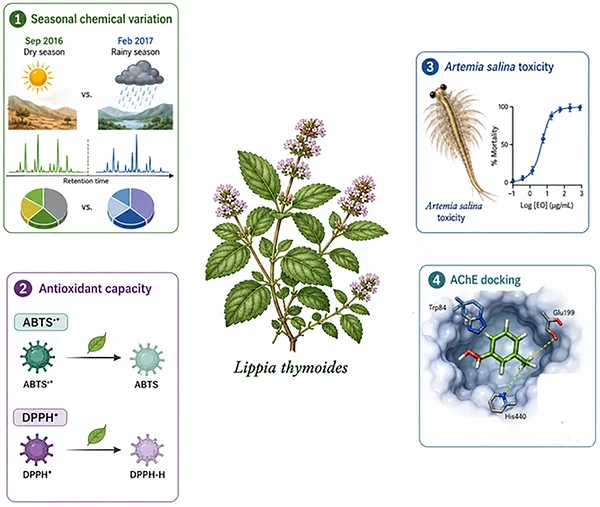

## 1 Introduction

Natural products remain an important source of bioactive compounds with potential applications in pharmacology, toxicology, food preservation, cosmetics, and biotechnology. Among these products, essential oils are particularly relevant because they are chemically complex volatile mixtures with broad biological potential. These volatile mixtures are mainly composed of monoterpenes, sesquiterpenes, and oxygenated derivatives, whose relative abundance may vary according to species, seasonality, plant organ, environmental conditions, and post‐harvest processing [1]. Therefore, chemical characterization is a fundamental step for interpreting the biological potential of essential oils and for identifying the compounds that may contribute to their pharmacological or toxicological profile [2, 3].

Oxidative stress results from an imbalance between the production of reactive species and the ability of biological systems to neutralize them. This process is associated with lipid peroxidation, protein oxidation, DNA damage, inflammation, cellular dysfunction, and the progression of several pathological conditions [4]. In this context, natural antioxidants have been investigated as alternatives or complements to synthetic compounds, particularly because plant‐derived secondary metabolites may act as radical scavengers, reducing agents, or modulators of oxidative processes [5]. Essential oils containing phenolic monoterpenes, such as thymol and carvacrol, have been frequently associated with antioxidant activity in vitro, although this activity depends on the chemical profile of each oil and on the experimental method used for evaluation [6].

The genus *Lippia* L. (Verbenaceae) comprises aromatic species widely distributed in tropical and subtropical regions and traditionally used in folk medicine, food, ornamentation, and other ethnobotanical practices. Several *Lippia* species produce essential oils rich in bioactive terpenoids and phenolic monoterpenes, which have been associated with antimicrobial, antioxidant, anti‐inflammatory, sedative, antispasmodic, insecticidal, and toxicological effects [7]. This chemical and biological diversity makes the genus a relevant source of essential oils for studies that combine phytochemical characterization with biological and computational approaches [8, 9].

*Lippia thymoides* Mart. & Schauer (Verbenaceae) is an aromatic species popularly known as “alecrim de cheiro miúdo,” “alecrim do mato,” and “alecrim do campo,” and in the Amazon region, particularly in Abaetetuba, Pará, as “manjerona” [10]. Previous studies have reported that *L. thymoides* Mart. & Schauer (Verbenaceae) essential oil (LTEO) may be characterized by a thymol‐rich profile, with other constituents such as p‐cymene, γ‐terpinene, thymol acetate, and sesquiterpenes contributing to its chemical variability [11, 12, 13]. Studies with this species have also indicated that seasonal variation, circadian rhythm, drying, and other processing conditions can influence essential oil yield, composition, and antioxidant activity, reinforcing the importance of evaluating the chemical profile of samples obtained under defined collection and processing conditions [11].

In addition to antioxidant assays, preliminary toxicity models are useful for estimating the biological activity and general toxicological profile of natural products. The *Artemia salina* lethality assay is widely used as a simple, low‐cost, and reproducible screening method for evaluating the general toxicity of plant extracts, essential oils, and isolated compounds [14, 15]. Although this model does not replace cytotoxicity or in vivo toxicological assays, it provides an initial indication of concentration‐dependent toxicity and may help guide subsequent biological investigations [15]. In studies involving essential oils, this assay is particularly relevant because volatile terpenoids can exert both pharmacological and toxicological effects depending on their chemical structure, concentration, and interaction with biological targets [16].

Molecular docking can complement experimental toxicity assays by predicting possible interactions between major oil constituents and molecular targets associated with biological responses. Acetylcholinesterase (AChE) has been explored as a target in studies involving essential oils and *A. salina* toxicity, since changes in cholinergic signaling may be associated with toxic responses in aquatic invertebrate models [17, 18]. However, docking results should be interpreted as predictive and complementary evidence, rather than definitive proof of mechanism [19, 20]. When integrated with chemical composition and experimental toxicity data, docking may help identify plausible molecular interactions that support a more mechanistic discussion of the observed biological effects [18, 21].

Although previous studies have substantially advanced the knowledge of LTEO chemistry [12, 13, 22, 23, 24], seasonal and circadian variation, antioxidant activity, and AChE‐related interactions, the relationship among collection‐period‐related chemical differences, antioxidant capacity, preliminary toxicity in *A. salina*, and predicted molecular interactions with AChE remains insufficiently integrated in a single experimental framework. This integration is important because the biological activity of essential oils may reflect not only the presence of a major compound, but also the contribution of minor constituents and the balance among chemical classes [16]. Therefore, the present study evaluated the chemical composition, antioxidant activity, preliminary toxicity, and molecular interactions of thymol‐rich LTEO obtained from leaves collected in two defined collection periods corresponding to dry and rainy seasonal conditions. To this end, the essential oil was chemically characterized by gas chromatography, evaluated for antioxidant capacity using ABTS•^+^ and DPPH• radical scavenging assays, tested for preliminary toxicity in *A. salina*, and analyzed by molecular docking against AChE.

## 2 Materials and Methods

### 2.1 Phytochemical Studies

#### 2.1.1 Botanical Material

Leaves of *L. thymoides* Mart. & Schauer (Verbenaceae) were collected in Abaetetuba, Pará, Brazil, in September 2016 and February 2017, corresponding to the dry and rainy seasonal periods, respectively. The botanical material was taxonomically identified by Carlos Alberto. A voucher specimen was deposited in the João Murça Pires Herbarium of the Museu Paraense Emílio Goeldi, Belém, Pará, Brazil, as part of the Aromatic Plants of the Amazon collection, under the registration number MG185797.

#### 2.1.2 Preparation and Characterization of Botanical Material

The leaves were dried in a forced‐air circulation oven at 35°C for 5 days. Subsequently, they were ground in a knife mill, homogenized, weighed, and subjected to the extraction process.

#### 2.1.3 Determination of Moisture Content

The moisture content of the samples was measured using an infrared moisture analyzer.

#### 2.1.4 EO Extraction

Each sample was subjected to hydrodistillation for 3 h using a modified Clevenger‐type glass apparatus connected to a cooling system to maintain the condensation water at approximately 12°C. After extraction, the essential oil was centrifuged at 3000 rpm for 5 min, dehydrated with anhydrous sodium sulfate, and subsequently centrifuged again under identical conditions. The oil was then stored in an amber glass ampoule, flame‐sealed, and kept refrigerated at 5°C [25].

#### 2.1.5 Calculation of the Yield of EO

The extraction yield (%) of the EO was determined based on the dry, moisture‐free raw material (dry mass content, DMC).

The gross yield of the essential oil was calculated by relating the volume of EO obtained to the mass of plant material used, according to Equation (1):

(1) $$\mathrm{Gross}\;\mathrm{oil}\;\mathrm{yield}=\frac{\mathrm{volume}\;\mathrm{of}\;\mathrm{oil}\;\mathrm{obtained}\;\left(\mathrm{mL}\right)}{\mathrm{mass}\;\mathrm{of}\;\mathrm{plant}\;\mathrm{material}\;\left(g\right)}\;\times 100$$

In which “volume of oil obtained (mL)” corresponds to the total volume of EO recovered after hydrodistillation, and “mass of plant material (*g*)” corresponds to the mass of *L. thymoides* Mart. & Schauer (Verbenaceae) leaves used in the extraction process.

The calculation of the moisture‐free basis (BLU) oil yield was performed by correcting the mass of the plant material as a function of its moisture content, according to Equation (2):

(2) $$\mathrm{Oil}\;\mathrm{BLU}=\frac{\mathrm{volume}\;\mathrm{of}\;\mathrm{oil}\;\mathrm{obtained}\;\left(\mathrm{mL}\right)}{m\;\left(g\right)-\left(\frac{m\left(g\right)\;\times \;U}{100}\right)}\;\times 100$$

In which m(g) represents the total mass of plant material initially used, and U represents the moisture content of the plant material. The term $\left(\frac{m\times U}{100}\right)$ corresponds to the mass of water present in the leaves, such that $\left[m(\frac{m\times U}{100})\right]$ reflects the effective dry mass used for calculating the dry‐basis yield.

#### 2.1.6 Analysis of the Chemical Composition of EO

The chemical composition of the essential oil was analyzed using a Shimadzu QP‐2010 double system comprising gas chromatography–mass spectrometry (GC–MS) and gas chromatography–flame ionization detection (GC–FID), Shimadzu, Kyoto, Japan. The operational conditions were consistent with previously established procedures, except for the use of hydrogen as the carrier gas. The retention index for all volatile constituents was calculated using a homologous series of n‐alkanes (C8–C40) from Sigma‐Aldrich (St. Louis, USA), following the method proposed by Van den Dool and Kratz [26]. Component identification was achieved by comparing the experimental mass spectra with those compiled in reference libraries and by correlating their retention indices with values reported in the literature [27].

### 2.2 In Vitro Antioxidant Capacity of the *LTEO*

The 2,2′‐azino‐bis (3‐ethylbenzothiazoline‐6‐sulfonic acid) radical cation (ABTS•^+^) and 2,2‐diphenyl‐1‐picrylhydrazyl (DPPH•) scavenging assays were employed to evaluate the antioxidant capacity of the essential oil, determined as Trolox equivalent antioxidant capacity (TEAC) using Trolox (6‐hydroxy‐2,5,7,8‐tetramethylchroman‐2‐carboxylic acid), a water‐soluble synthetic analogue of vitamin E. Both ABTS•^+^ and DPPH• assays were performed in three independent determinations for each LTEO sample (*n* = 3 per sample per assay).

#### 2.2.1 ABTS•+ Assay

The ABTS•^+^ radical scavenging assay was performed according to the methodology adapted from Miller et al. [28] and modified by Re et al. [29]. The ABTS•^+^ solution was prepared using 7 mM ABTS and 140 mM potassium persulfate (K_2_O_8_S_2_), incubated at room temperature, and protected from light for 16 h. The resulting solution was then diluted with phosphate‐buffered saline until reaching an absorbance of 0.700 ± 0.02 at 734 nm. To measure the antioxidant capacity, 2.97 mL of the ABTS•^+^ solution were transferred to a cuvette, and the absorbance at 734 nm was recorded using a Biospectro SP 22 spectrophotometer. Subsequently, 0.03 mL of the sample was added to the cuvette containing the ABTS•^+^ radical, and after 5 min, a second reading was performed. Trolox was used as the standard solution for the calibration curve.

#### 2.2.2 DPPH• Assay

The assay was performed according to the method proposed by Blois [30]. To assess the antioxidant capacity, the absorbance of a 0.1 mM DPPH• solution diluted in ethanol was first determined. Subsequently, 0.6 mL of the DPPH• solution, 0.35 mL of distilled water, and 0.05 mL of the sample were mixed and placed in a water bath at 30°C for 30 min. After incubation, the absorbance was measured at 517 nm using a Biospectro SP 22 spectrophotometer. The synthetic antioxidant Trolox was used as the standard solution for the calibration curve.

### 2.3 Preliminary Toxicity Bioassay With *A. salina* Leach

The preliminary toxicity bioassay was performed using LTEO samples obtained from the September 2016 and February 2017 collections. The lethality assay with *A. salina* Leach was conducted according to a previously described method [14]. Each LTEO sample was tested at concentrations of 1, 5, 10, 25, 50, and 100 µg mL^−^^1^. Artificial seawater and DMSO were used as the solvent system at a 95:5 ratio. Ten *A. salina* nauplii were transferred to each test flask. The negative control contained only the solvent system, without essential oil, whereas lapachol was used as the positive control under the same experimental conditions. After 24 h of exposure, dead nauplii were counted at each concentration, and the mortality rate was calculated. The LC_50_ value was estimated by Probit analysis. Confidence intervals for LC_50_ values were not available from the original Probit analysis output; therefore, LC_50_ values are reported as mean ± standard deviation (SD). For each LTEO sample and each tested concentration, the assay was performed in triplicate (*n* = 3), with 10 *A. salina* nauplii per replicate, totaling 30 nauplii per concentration.

### 2.4 In Silico Evaluation of Major EO Components

However, this approach was considered predictive and hypothesis‐generating, since no experimental AChE inhibition assay was performed. AChE was selected as the molecular target because the *A. salina* lethality assay was used in this study as a preliminary toxicity screening model, and cholinesterase‐related pathways may represent plausible neurotoxic mechanisms in aquatic invertebrates exposed to bioactive natural products. The antioxidant assays performed in this study, ABTS•^+^ and DPPH•, were used to evaluate chemical radical‐scavenging capacity and were not intended to indicate direct cholinergic or AChE‐related effects. In contrast, the *A. salina* lethality assay provided a preliminary biological toxicity endpoint, for which AChE was considered a relevant exploratory target because cholinergic disruption has been associated with toxic responses in invertebrate models. Therefore, docking LTEO constituents against AChE was used as a complementary computational approach to explore whether compounds present in the essential oil could interact with a biologically relevant enzymatic target potentially associated with the lethality response observed in *A. salina* [17, 18, 31]. However, this approach was considered predictive and hypothesis‐generating, since no experimental AChE inhibition assay was performed. The major compounds identified in LTEO, thymol, p‐cymene, γ‐terpinene, thymol acetate, (E)‐caryophyllene, and myrcene, were used as models for interaction. The three‐dimensional structure of the AChE protein was obtained from the Protein Data Bank with the PDB ID 6XYU [32]. This crystallographic structure was selected because it contains a well‐defined ligand‐binding region and a co‐crystallized ligand, allowing validation of the docking protocol by re‐docking. The substances used in our studies were retrieved from PubChem, and the molecular structures of these compounds were optimized using the B3LYP/6‐31G method [33, 34] with Gaussian 09 [35]. To evaluate the molecular binding mode, we employed Molegro Virtual Docker 5.5 software [36]. Before docking the LTEO constituents, the docking protocol was validated by re‐docking the co‐crystallized ligand into the AChE binding pocket. For this procedure, the crystallographic ligand was removed from the protein structure and subsequently docked back into the same binding site using the same docking parameters applied to the LTEO constituents. The accuracy of the protocol was evaluated by calculating the root‐mean‐square deviation (RMSD) between the redocked pose and the original crystallographic pose. The re‐docking procedure yielded an RMSD value of 1.7 Å, indicating adequate reproduction of the experimental binding pose and supporting the reliability of the docking protocol. The MolDock Score (GRID) scoring function was utilized with a grid resolution of 0.30 Å and a radius of 5 Å encompassing the entire binding cavity. The MolDock SE algorithm was employed for docking with 10 runs, a maximum of 1500 iterations, and a maximum population size of 50. The molecular docking simulation used a maximum evaluation of 300 steps with a neighbor distance factor of 1 and an energy threshold of 100.

### 2.5 Statistical Analysis

Chemical composition data were expressed as relative percentages of the total essential oil composition. Antioxidant capacity data were expressed as mean ± standard deviation (SD) from three independent determinations (*n* = 3). For the preliminary toxicity assay, each concentration was tested in triplicate, with 10 *A. salina* nauplii per replicate, and mortality data were expressed as percentages after 24 h of exposure. LC_50_ values were estimated by Probit analysis and are presented as mean ± standard deviation (SD). Data were analyzed on their original scale, without transformation or normalization. The datasets were inspected for possible data entry errors and outliers, and no values were excluded from the analyses. No inferential statistical comparisons were performed between the September 2016 and February 2017 samples; therefore, no *p* values or significance symbols are presented. Differences between samples were interpreted descriptively.

## 3 Results

### 3.1 Essential Oil Yield

The essential oil yield obtained from *L. thymoides* Mart. & Schauer (Verbenaceae) leaves varied between the two collection periods. The September/2016 sample yielded 2.24% on a moisture‐free basis, whereas the February/2017 sample yielded 2.70%. Thus, the highest essential oil yield was recorded for the material collected in February 2017.

### 3.2 Chemical Composition of LTEO

Table 1 summarizes the chemical constituents identified in LTEO obtained from leaves collected in September 2016 and February 2017, listed according to their retention indices. A total of 38 constituents were identified across the two samples.

**TABLE 1:** Chemical constituents identified in the LTEO.

RI(C)	RI(L)	Constituents	September/2016 (%)	February/2017 (%)
922	924	α‐Thujene	1.84	3.95
969	969	Sabinene	—	0.06
976	974	1‐Octen‐3‐ol	—	0.41
987	988	Myrcene	4.30	6.40
1008	1002	α‐Phellandrene	0.55	0.58
1017	1014	α‐Terpinene	3.11	4.16
1026	1020	*p*‐Cymene	11.10	20.41
1062	1054	γ‐Terpinene	9.63	19.97
1070	1065	cis‐Sabinene hydrate	—	0.16
1084	1088	Terpinolene	0.32	0.19
1090	1089	*p*‐Cymenene	0.23	0.07
1101	1095	Linalool	0.34	0.24
1151	1141	Camphor	0.62	—
1169	1167	Umbellulone	—	0.49
1188	1174	Terpinen‐4‐ol	—	0.76
1230	1232	Thymol methyl ether	3.61	1.78
1305	1289	Thymol	32.13	30.41
1355	1349	Thymol acetate	7.82	4.40
1358	1356	Eugenol	0.20	0.09
1367	1370	Carvacrol acetate	0.04	—
1376	1374	α‐Copaene	0.20	—
1423	1417	(E)‐Caryophyllene	7.26	3.90
1429	1414	γ‐Elemene	0.43	0.05
1433	1432	trans‐α‐Bergamotene	0.66	0.09
1449	1442	6,9‐Guaiadiene	0.11	—
1456	1452	α‐Humulene	2.27	0.54
1474	1478	γ‐Muurolene	0.71	0.08
1477	1484	Germacrene D	0.67	0.46
1497	1500	α‐Muurolene	0.31	—
1501	1502	trans‐β‐Guaiene	0.14	—
1512	1513	γ‐Cadinene	0.58	0.05
1517	1522	δ‐Cadinene	0.91	0.13
1521	1521	trans‐Calamenene	0.10	—
1523	1521	β‐Sesquiphellandrene	0.07	—
1531	1533	trans‐Cadina‐1,4‐diene	0.09	—
1535	1537	α‐Cadinene	0.13	—
1581	1582	Caryophyllene oxide	1.21	0.17
1655	1652	α‐Cadinol	0.02	—

Abbreviations: RI(C), calculated retention index; RI(L): literature retention index obtained from Adams [27].

Thymol was the major constituent of LTEO in both collection periods, accounting for 32.13% in September 2016 and 30.41% in February 2017. Among the other major constituents, p‐cymene and γ‐terpinene showed higher relative abundances in the February sample, increasing from 11.00% and 9.63% in September 2016 to 20.41% and 19.97% in February 2017, respectively. In contrast, thymol acetate and (E)‐caryophyllene were more abundant in the September sample, corresponding to 7.82% and 7.26%, respectively, compared with 4.40% and 3.90% in February 2017.

The distribution of chemical classes varied between the two collection periods. In September 2016, oxygenated monoterpenes represented the predominant class, accounting for 44.56% of the oil, followed by monoterpene hydrocarbons (30.87%) and sesquiterpene hydrocarbons (14.64%). In February 2017, monoterpene hydrocarbons were the predominant class, corresponding to 55.95%, followed by oxygenated monoterpenes (38.08%) and sesquiterpene hydrocarbons (5.30%).

**FIGURE 1:**
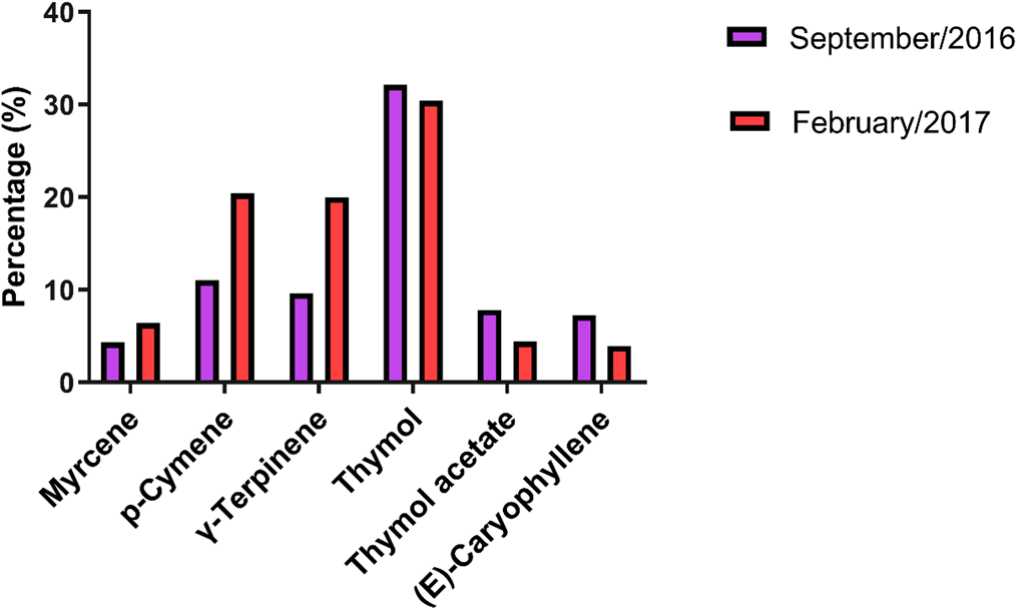
Collection‐period‐related variation in the relative abundance of the major essential oil constituents collected in September 2016 and February 2017. Values are expressed as relative percentages of the total essential oil composition. No inferential statistical analysis was applied.

The chemical profile varied between the two collection periods, with changes in the relative abundance of the main constituents (Figure 1). Thymol was the most abundant compound in both September 2016 and February 2017, accounting for 32.13% and 30.41% of the total composition, respectively. Although thymol remained the predominant constituent, its relative abundance was slightly lower in February 2017.

Among the monoterpene hydrocarbons, p‐cymene increased from 11.00% in September 2016 to 20.41% in February 2017, while γ‐terpinene increased from 9.63% to 19.97% over the same period. Myrcene also showed a higher relative abundance in February 2017, increasing from 4.30% to 6.40%.

In contrast, thymol acetate decreased from 7.82% in September 2016 to 4.40% in February 2017. A similar reduction was observed for (E)‐caryophyllene, which declined from 7.26% to 3.90% between the two collection periods. Overall, the February 2017 sample contained higher proportions of monoterpene hydrocarbons, whereas some oxygenated and sesquiterpenic constituents were more abundant in September 2016.

**FIGURE 2:**
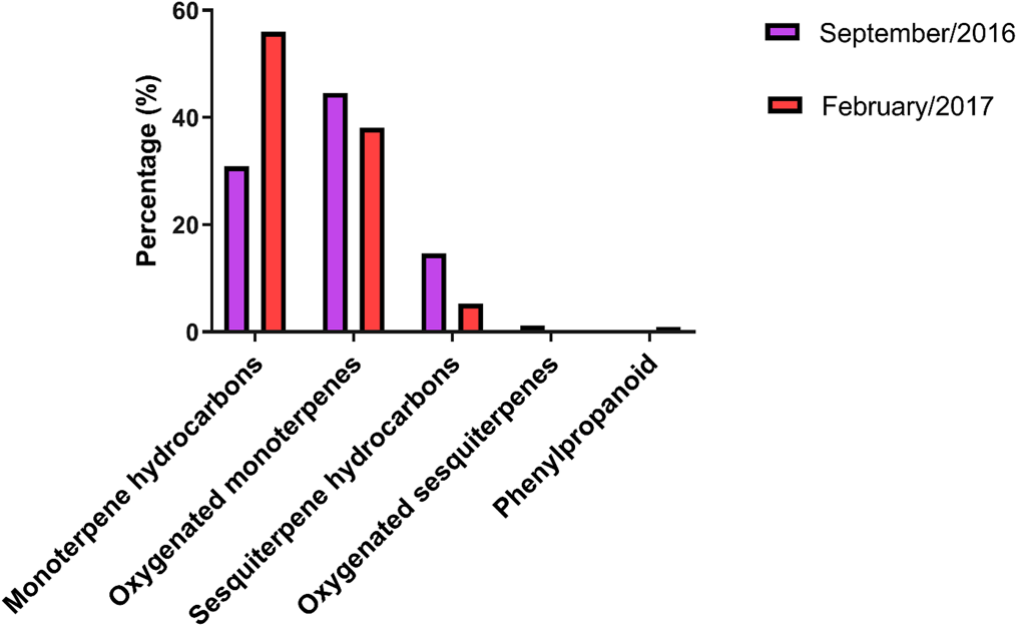
Distribution of chemical classes in the essential oil samples collected in September 2016 and February 2017. Values are expressed as relative percentages of the total essential oil composition. No inferential statistical analysis was applied.

The distribution of chemical classes also differed between September 2016 and February 2017 (Figure 2). In September 2016, oxygenated monoterpenes represented the predominant class, corresponding to 44.56% of the chemical profile, followed by monoterpene hydrocarbons at 30.87% and sesquiterpene hydrocarbons at 14.64%. Oxygenated sesquiterpenes and phenylpropanoids were detected at lower relative proportions, representing 1.23% and 0.20%, respectively.

In February 2017, monoterpene hydrocarbons became the predominant chemical class, increasing to 55.95%. Oxygenated monoterpenes accounted for 38.08%, while sesquiterpene hydrocarbons decreased to 5.30%. Oxygenated sesquiterpenes were detected at 0.17%, and phenylpropanoids represented 0.09% of the total composition. These data indicate a shift in the chemical class distribution between the two sampling periods, characterized mainly by an increased proportion of monoterpene hydrocarbons in February 2017 and a lower relative contribution of sesquiterpene classes.

### 3.3 In Vitro Antioxidant Activity

**FIGURE 3:**
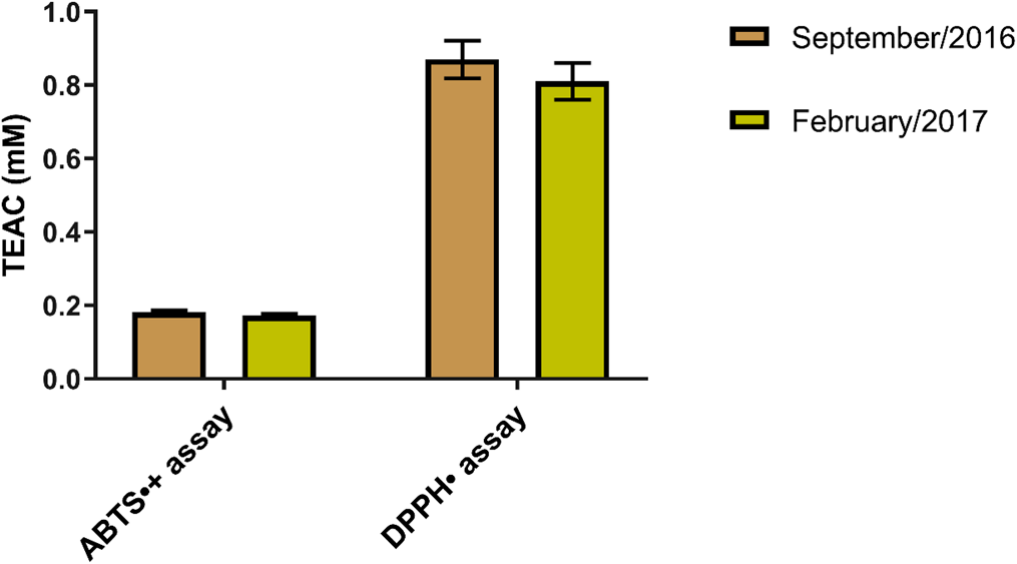
Trolox equivalent antioxidant capacity of LTEO assessed by ABTS•^+^ and DPPH• radical scavenging assays. Values are expressed as mean ± standard deviation (SD) from three independent determinations (*n* = 3). No inferential statistical analysis was applied; therefore, no *p* values or significance symbols are shown.

The Trolox equivalent antioxidant capacity (TEAC) of LTEO was assessed using ABTS•^+^ and DPPH• radical scavenging assays, as shown in Figure 3. In the ABTS•^+^ assay, both samples displayed similar TEAC values, with 0.181 ± 0.006 mM for September 2016 and 0.172 ± 0.006 mM for February 2017.

The DPPH• assay yielded higher TEAC values than the ABTS•^+^ assay for both collection periods. The September 2016 sample reached 0.870 ± 0.051 mM, whereas the February 2017 sample reached 0.810 ± 0.050 mM. In both antioxidant assays, the September 2016 sample had numerically higher TEAC values than the February 2017 sample.

### 3.4 Preliminary Toxicity Bioassay in *A. salina*

The preliminary toxicity assay indicated that both LTEO samples induced concentration‐dependent mortality in *A. salina*, with detectable lethality beginning at 10 µg mL^−^^1^. These results are summarized in Table 2.

**TABLE 2:** Preliminary toxicity of LTEO samples collected in September 2016 and February 2017 against *A. salina*.

Sample	Concentration (µg mL^−^^1^)	Mortality rate (%)	LC_50_ (µg mL^−^^1^)
LTEO September 2016	100	60	71.08 ± 7.06
	50	50	
	25	26.67	
	10	10	
	5	0	
	1	0	
LTEO February 2017	100	63.33	60.06 ± 6.12
	50	53.33	
	25	30.00	
	10	13.33	
	5	0	
	1	0	

*Note*: Values are expressed as mortality percentage and LC_50_ mean ± standard deviation (SD). Each concentration was tested in triplicate, with 10 *A. salina* nauplii per replicate. LC_50_ values were estimated by Probit analysis.

For the September 2016 LTEO sample, mortality increased from 10% at 10 µg mL^−^^1^ to 26.67% at 25 µg mL^−^^1^ and 50% at 50 µg mL^−^^1^, reaching 60% at the highest tested concentration of 100 µg mL^−^^1^. The estimated LC_50_ for this sample was 71.08 ± 7.06 µg mL^−^^1^, expressed as mean ± SD.

A similar concentration‐dependent pattern was observed for the February 2017 LTEO sample. Mortality increased from 13.33% at 10 µg mL^−^^1^ to 30.00% at 25 µg mL^−^^1^ and 53.33% at 50 µg mL^−^^1^, reaching 63.33% at 100 µg mL^−^^1^. The estimated LC_50_ for this sample was 60.06 ± 6.12 µg mL^−^^1^, expressed as mean ± SD.

The February 2017 sample showed a numerically lower LC_50_ than the September 2016 sample, together with slightly higher mortality rates at the concentrations that produced detectable lethality.

### 3.5 Molecular Docking

Molecular docking was performed to evaluate the predicted binding of the major LTEO constituents to the AChE binding pocket (Table 3). The dimensionless MolDock Score values ranged from −75.03 to −101.21, indicating differences in the predicted interaction profile among the evaluated ligands. Among the tested compounds, (E)‐caryophyllene exhibited the most favorable MolDock Score, with a value of −101.21, followed by thymol acetate (−98.51) and myrcene (−84.61). Thymol showed an intermediate score of −79.83, whereas p‐cymene and γ‐terpinene had the least favorable values, with MolDock Scores of −76.94 and −75.03, respectively.

**TABLE 3:** MolDock score values of major LTEO constituents docked into the AChE binding pocket.

Ligands	MolDock score
Thymol	−79.83
p‐Cymene	−76.94
γ‐Terpinene	−75.03
Thymol acetate	−98.51
(E)‐Caryophyllene	−101.21
Myrcene	−84.61

*Note*: MolDock Score is a dimensionless computational docking score and therefore has no unit of measurement. MolDock Score values are computational docking scores and were not subjected to inferential statistical analysis.

**FIGURE 4:**
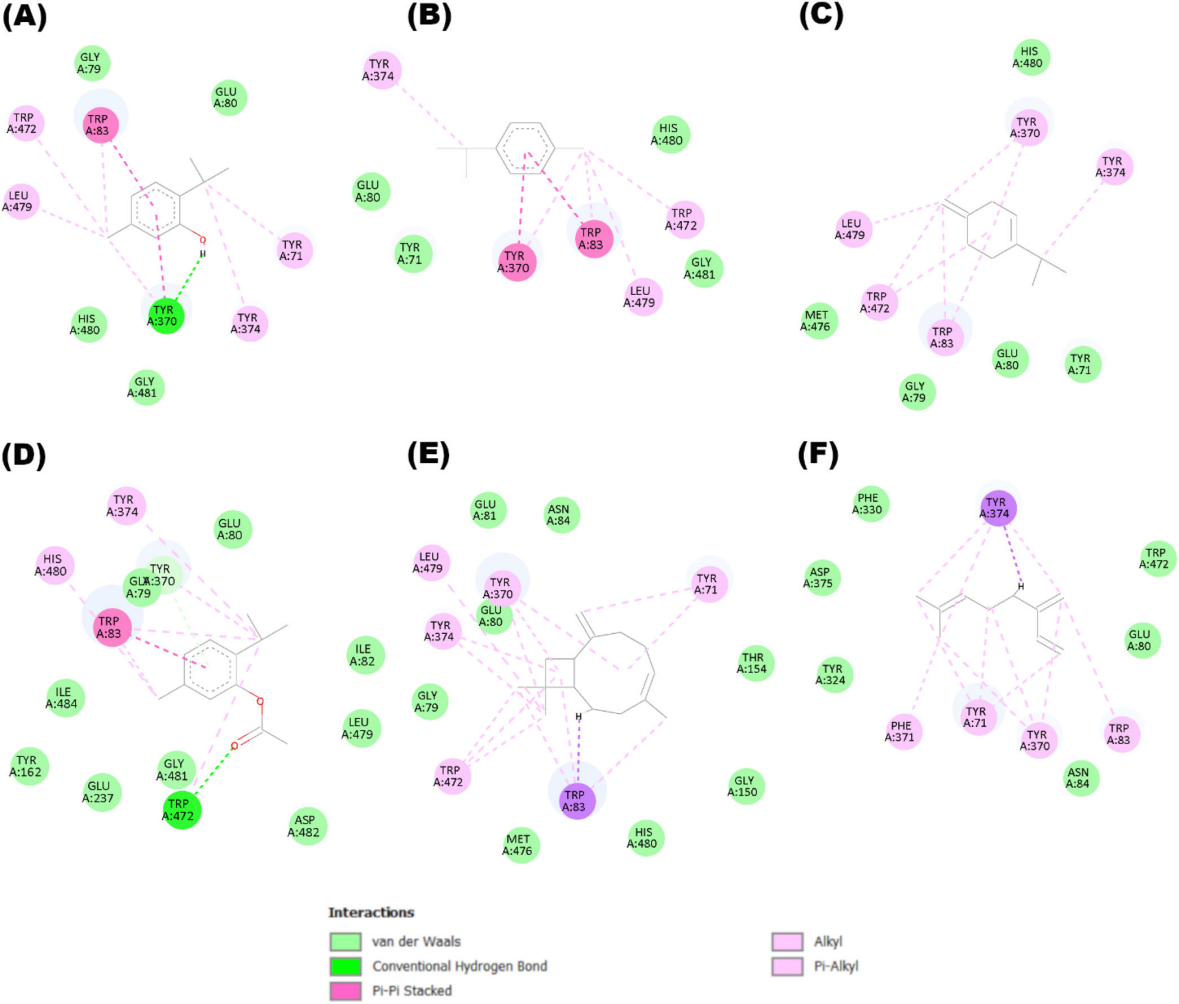
Two‐dimensional interaction profiles of major LTEO constituents docked into the AChE binding pocket. (A) Thymol; (B) p‐cymene; (C) γ‐terpinene; (D) thymol acetate; (E) (E)‐caryophyllene; (F) myrcene. The figure represents predicted ligand–protein interaction maps obtained from molecular docking; no inferential statistical analysis was applied.

The two‐dimensional interaction profiles of the major LTEO constituents within the AChE binding pocket are displayed in Figure 4.

The ligands interacted with residues located in the binding region mainly through hydrophobic contacts, including alkyl and π‐alkyl interactions, together with van der Waals contacts. Conventional hydrogen bonds were observed only for thymol and thymol acetate.

Thymol interacted with the AChE binding pocket through a conventional hydrogen bond with Tyr370. In addition, this ligand established hydrophobic and π‐associated contacts with aromatic and nonpolar residues, including Trp83, Tyr370, Tyr71, Tyr374, Trp472, and Leu479, while Gly79, Glu80, His480, and Gly481 contributed through van der Waals contacts (Figure 4A).

p‐Cymene was accommodated in the binding pocket predominantly through hydrophobic interactions. The interaction map indicated contacts with Trp83, Tyr370, Leu479, and Trp472, whereas Tyr71, Glu80, His480, and Gly481 were positioned around the ligand through van der Waals interactions (Figure 4B).

γ‐Terpinene also displayed a hydrophobic interaction profile, with contacts involving Trp83, Tyr370, Tyr374, Trp472, and Leu479. Additional residues, including Met476, Gly79, Glu80, Tyr71, and His480, were located around the ligand through van der Waals interactions (Figure 4C).

Thymol acetate interacted with the AChE binding pocket through a conventional hydrogen bond with Trp472. The interaction profile also included hydrophobic contacts involving Trp83, Tyr370, Tyr374, and His480. Other residues, including Gly79, Glu80, Gly481, Leu479, Ile484, Tyr162, Glu237, and Asp482, contributed to the ligand environment through van der Waals contacts (Figure 4D).

(E)‐Caryophyllene exhibited an interaction profile dominated by hydrophobic contacts. The ligand interacted with residues such as Trp83, Tyr370, Tyr374, Tyr71, Trp472, and Leu479, while Glu80, Glu81, Asn84, Gly79, Met476, His480, Thr154, and Gly150 were positioned around the ligand through van der Waals interactions (Figure 4E).

Myrcene interacted with the AChE binding pocket mainly through hydrophobic contacts involving Tyr374, Phe371, Tyr71, Tyr370, and Trp83. Additional residues, including Phe330, Asp375, Tyr324, Trp472, Glu80, Asn84, and Gly150, were observed in the surrounding interaction environment through van der Waals contacts (Figure 4F).

**FIGURE 5:**
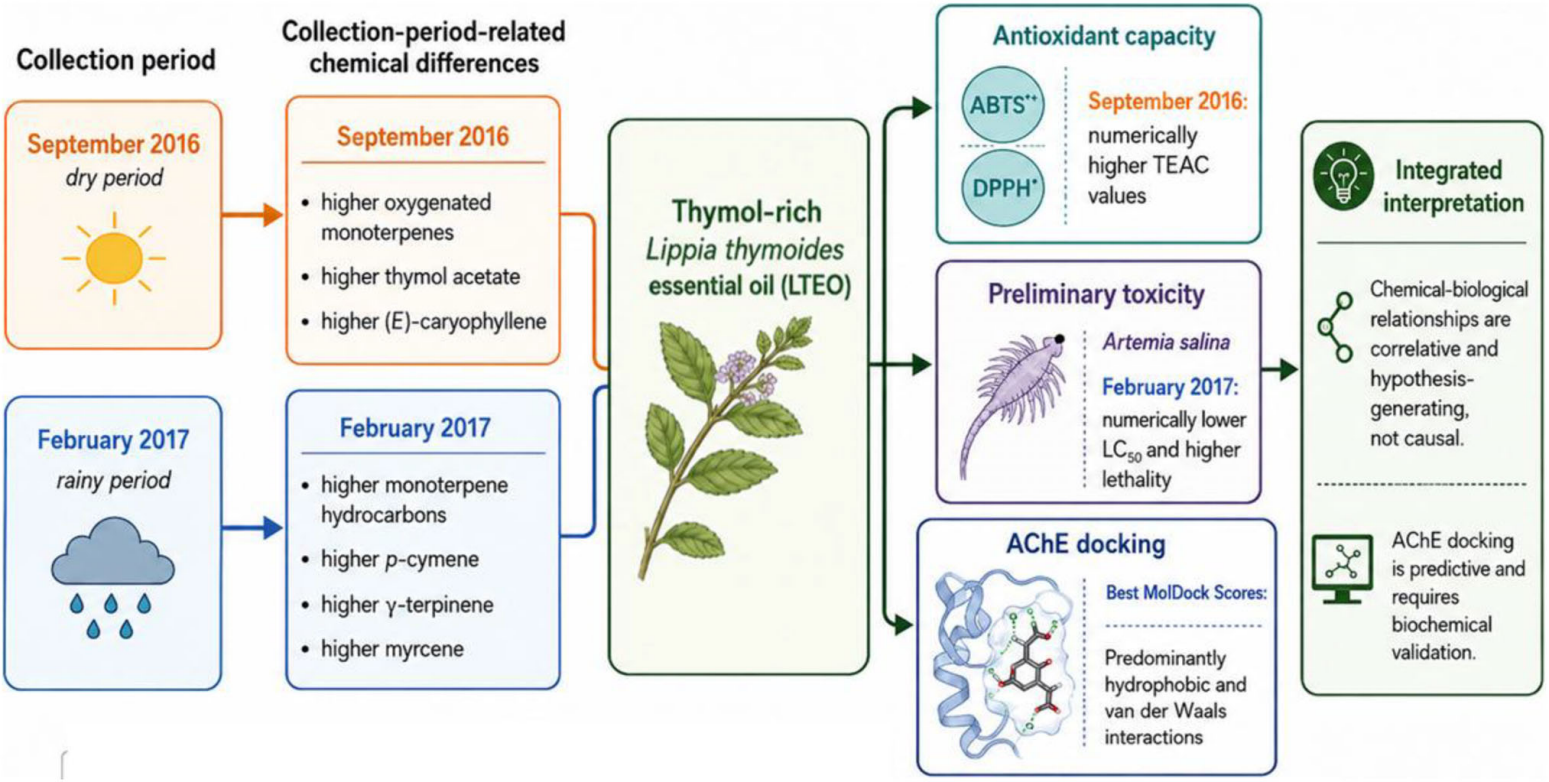
Schematic overview of the integrated relationships among collection period, chemical composition, antioxidant capacity, *Artemia salina* lethality, and AChE docking results in *Lippia thymoides* essential oil (LTEO). The September 2016 and February 2017 samples are shown as thymol‐rich oils with collection‐period‐related differences in chemical class distribution, particularly in oxygenated monoterpenes, monoterpene hydrocarbons, and sesquiterpenes. These chemical differences are descriptively associated with variations in antioxidant capacity and preliminary lethality in *A. salina*. Docking results are presented as predictive molecular evidence of possible interactions between major LTEO constituents and the AChE binding pocket. The relationships shown in this scheme are correlative and hypothesis‐generating, not causal.

A schematic overview summarizing the relationships among collection period, chemical composition, antioxidant capacity, *A. salina* lethality, and AChE docking results is presented in Figure 5.

## 4 Discussion

This study investigated the seasonal chemical variation, antioxidant capacity, preliminary toxicity, and AChE docking profile of thymol‐rich *L. thymoides* Mart. & Schauer (Verbenaceae) essential oil. By integrating phytochemical characterization with in vitro radical‐scavenging assays, *A. salina* lethality, and molecular docking, the study addressed a relevant gap in the characterization of LTEO: the need to relate seasonal variation in volatile composition to complementary biological and computational endpoints. Overall, LTEO retained a thymol‐rich profile in both collection periods, but differed quantitatively in yield, chemical class distribution, antioxidant response, preliminary lethality, and predicted interaction with AChE.

The phytochemical data confirmed that *L. thymoides* Mart. & Schauer (Verbenaceae) produces a thymol‐type essential oil, in agreement with previous reports for this species [12, 13, 23]. Thymol remained the predominant constituent in both seasonal samples, indicating that this phenolic monoterpene is a consistent chemical marker of the analyzed LTEO. However, the two samples differed in yield and in the relative abundance of major and intermediate‐abundance constituents. The February 2017 sample produced the highest oil yield, whereas the September 2016 sample contained a slightly higher thymol content and a greater proportion of oxygenated monoterpenes. These differences support the interpretation that the core chemical identity of LTEO was conserved, while its quantitative profile was modulated by the collection period.

Seasonal and environmental factors are known to influence essential oil biosynthesis, affecting both oil accumulation and the relative distribution of volatile constituents [23, 37]. In the present study, this variability was reflected in the shift between oxygenated monoterpenes and monoterpene hydrocarbons. The September 2016 sample was characterized by a higher proportion of oxygenated monoterpenes, whereas the February 2017 sample was enriched in monoterpene hydrocarbons, mainly p‐cymene, γ‐terpinene, and myrcene. Because p‐cymene and γ‐terpinene are chemically related to thymol‐rich profiles, their increased abundance in February may indicate a seasonal redistribution within the monoterpene fraction rather than a complete change in chemotype.

It is important to note that this study was based on two specific collection periods, September 2016 and February 2017, corresponding to dry and rainy seasonal conditions, respectively. Therefore, the observed differences should be interpreted as collection‐period‐related variation rather than as evidence of a complete annual seasonal pattern. Broader conclusions about seasonal dynamics in LTEO would require additional sampling across multiple months, years, and environmental conditions.

The sesquiterpene fraction also contributed to the chemical differentiation between the samples. The higher relative abundance of (E)‐caryophyllene and other sesquiterpene hydrocarbons in September 2016 distinguished this sample from the February 2017 oil. Although these constituents were less abundant than thymol, p‐cymene, and γ‐terpinene, they may still influence the global physicochemical and biological profile of the essential oil mixture. This interpretation is consistent with the general concept that essential oil bioactivity may depend not only on the major compound, but also on the balance among chemical classes and the contribution of constituents present at intermediate or minor levels. Nevertheless, because isolated compounds were not tested individually, the role of specific constituents should be interpreted as a plausible correlative association rather than a demonstrated causal effect. Therefore, the chemical differences observed between the two LTEO samples should not be considered direct evidence that any specific compound or chemical class caused the antioxidant or toxicological responses.

The antioxidant assays indicated that LTEO exhibited in vitro radical‐scavenging capacity in both ABTS•^+^ and DPPH• systems, with higher TEAC values in the DPPH• assay. This difference is expected because ABTS•^+^ and DPPH• assays rely on distinct radical systems and may respond differently to essential oil constituents depending on polarity, solubility, steric accessibility, reaction kinetics, and chemical structure [7, 29]. Thus, the antioxidant profile observed for LTEO should be understood as assay‐dependent rather than as a single fixed property of the oil.

Between the two samples, the September 2016 oil showed slightly higher TEAC values in both assays. This pattern is correlatively consistent with its higher proportion of oxygenated monoterpenes and slightly higher thymol content. Thymol is a phenolic monoterpene frequently associated with radical‐scavenging activity in essential oils, and its predominance in both LTEO samples provides a plausible chemical basis for the observed antioxidant response [23, 29]. However, because only the complete essential oil mixture was tested, the antioxidant response cannot be attributed exclusively to thymol or to any specific constituent. The possible contribution of thymol acetate, (E)‐caryophyllene, and other constituents remains correlative and requires confirmation using isolated compounds or recombined mixtures.

Conversely, the February 2017 sample, although richer in monoterpene hydrocarbons, showed slightly lower TEAC values. This finding suggests that enrichment in hydrocarbon monoterpenes did not translate into greater radical‐scavenging capacity under the experimental conditions used. Thus, chemical class distribution should be considered when interpreting antioxidant assays with essential oils. Even so, ABTS•^+^ and DPPH• are chemical assays and do not reproduce the complexity of biological oxidative stress. Therefore, the antioxidant activity observed here should be regarded as a preliminary chemical indication of radical‐scavenging capacity, not as evidence of antioxidant efficacy in cellular or in vivo systems.

The *A. salina* assay provided an initial and preliminary toxicological screening of LTEO. Both samples produced concentration‐dependent lethality, with no mortality at the two lowest concentrations and detectable lethality beginning at 10 µg mL^−^^1^. The February 2017 sample was numerically more toxic, as indicated by its lower LC_50_ and slightly higher mortality rates at concentrations that produced detectable lethality. This difference may be correlatively associated with the collection‐period‐related shift in chemical composition, particularly the higher relative abundance of monoterpene hydrocarbons in the February oil. However, the current data do not allow toxicity to be assigned to one compound or chemical class, nor do they demonstrate that the observed chemical differences caused the difference in lethality. Importantly, lethality in *A. salina* should not be interpreted as direct evidence of mammalian toxicity or as an indicator of human or animal safety.

Essential oils are complex mixtures, and their toxicity in *A. salina* may reflect combined effects of major, intermediate‐abundance, and minor constituents rather than the isolated action of a single molecule. In this context, the greater lethality of the February 2017 sample may be correlatively related to the overall balance among monoterpene hydrocarbons, oxygenated monoterpenes, and sesquiterpenes, but this association should not be interpreted as evidence of a causal relationship. The *A. salina* lethality assay is widely used as a simple and sensitive preliminary screening model for natural products [14], but it does not replace cytotoxicity assays, enzymatic tests, mammalian toxicity assays, or in vivo toxicological models. Therefore, the LC_50_ values obtained for LTEO should be interpreted only as indicators of preliminary lethality under the experimental conditions used, without extrapolation to mammalian toxicity, human safety, animal safety, or toxicity in higher organisms. Additional toxicological investigations would be required to determine whether LTEO presents relevant safety or toxicity concerns in mammalian, veterinary, or human contexts.

The biological relevance of selecting AChE as the docking target should be interpreted in the context of the experimental endpoints used in this study. The ABTS•^+^ and DPPH• assays provided information on the chemical radical‐scavenging capacity of LTEO, but these assays do not directly assess cholinergic signaling or AChE inhibition. In contrast, the *A. salina* assay provided a preliminary biological endpoint related to lethality in an aquatic invertebrate model. Because AChE is a key enzyme involved in cholinergic neurotransmission and has been investigated as a toxicity‐related target in studies with essential oils and invertebrate models, it was selected here as an exploratory molecular target to complement the *A. salina* lethality data. Nevertheless, this connection remains predictive and hypothesis‐generating, since the present study did not include a biochemical AChE inhibition assay.

The molecular docking analysis complemented the experimental toxicity data by evaluating the predicted interaction of major LTEO constituents with the AChE binding pocket as a possible toxicity‐related target rather than as a confirmed mechanism of action. Among the evaluated ligands, (E)‐caryophyllene and thymol acetate displayed the most favorable MolDock Score values, followed by myrcene, thymol, p‐cymene, and γ‐terpinene. This ranking is important because the best predicted interactions were not restricted to the most abundant constituents of LTEO. Although thymol was the dominant compound in both samples, (E)‐caryophyllene and thymol acetate showed more favorable docking scores, suggesting that constituents present at intermediate relative abundance may contribute substantially to the molecular interaction profile of the oil.

The interaction maps showed that the major LTEO constituents were accommodated within the AChE binding pocket mainly through hydrophobic contacts, including alkyl and π‐alkyl interactions, together with van der Waals contacts. This pattern is consistent with the nonpolar nature of many terpenoids and suggests that the evaluated ligands occupied a related region of the AChE binding pocket. Similar hydrophobic and π‐associated contacts have been described in molecular modeling studies involving essential oil constituents and AChE [13].

The two ligands with the most favorable docking scores displayed distinct interaction features. (E)‐Caryophyllene interacted mainly through hydrophobic contacts, whereas thymol acetate combined hydrophobic contacts with a conventional hydrogen bond involving Trp472. Although thymol was the major compound, its intermediate MolDock Score reinforces that predicted affinity was influenced by structural features, interaction type, and ligand fit within the AChE binding pocket rather than abundance alone.

The docking results also help frame, but do not prove, a possible molecular rationale for the toxicity observed in *A. salina*. AChE has been explored as a molecular target in studies involving essential oil toxicity and aquatic invertebrate models, and the ability of LTEO constituents to interact with the AChE binding pocket is consistent with a plausible cholinergic‐related hypothesis. However, docking is a predictive computational approach and does not demonstrate enzymatic inhibition, binding affinity under experimental conditions, AChE modulation, or a causal relationship with larval mortality. Importantly, no experimental AChE inhibition assay was performed in this study. Therefore, the docking results should be interpreted only as preliminary, hypothesis‐generating evidence, and no conclusion regarding AChE modulation can be drawn without biochemical validation. Functional AChE inhibition assays would be required to determine whether the predicted interactions translate into measurable enzyme modulation.

## 5 Conclusions

This study showed that *L. thymoides* Mart. & Schauer (Verbenaceae) essential oil maintained a thymol‐rich chemical profile between the two analyzed collection periods, while displaying quantitative variation in yield, major constituents, chemical classes, antioxidant capacity, preliminary toxicity, and predicted AChE interactions. The September 2016 sample was characterized by a higher proportion of oxygenated monoterpenes and slightly greater radical‐scavenging capacity, whereas the February 2017 sample showed higher oil yield, enrichment in monoterpene hydrocarbons, and greater preliminary lethality in *A. salina*. The integration of chemical, biological, and computational data indicates that LTEO bioactivity cannot be interpreted solely from its major compound but rather may be correlatively associated with the combined contribution of thymol, related monoterpenes, sesquiterpenes, and the collection‐period‐related balance among chemical classes. Molecular docking suggested that major and intermediate‐abundance constituents may interact with the AChE binding pocket, mainly through hydrophobic and van der Waals contacts, providing predictive and preliminary molecular support for future mechanistic investigations. The AChE docking endpoint should therefore be understood as an exploratory link to the preliminary *A. salina* toxicity assay, not as evidence of a direct antioxidant mechanism or experimentally confirmed AChE inhibition. Overall, this study advances the characterization of thymol‐rich *L. thymoides* Mart. & Schauer (Verbenaceae) essential oil under two defined collection conditions and supports its relevance as a chemically variable natural product with antioxidant potential, preliminary toxicological activity, and predicted interactions with a biologically relevant enzymatic target.

## Author Contributions

**Jorddy Neves Cruz**: conceptualization (lead), data curation (lead), formal analysis (lead), investigation (lead), methodology (lead), visualization (lead), writing – original draft preparation (lead), writing – review and editing (equal). **Sebastião Gomes Silva**: investigation (equal), methodology (equal), resources (equal), validation (equal), writing – review and editing (equal). **Angelo Antonio Barbosa Morais**: formal analysis (equal), methodology (equal), software (equal), validation (equal), writing – review and editing (equal). **Anderson Bentes de Lima**: investigation (equal), formal analysis (supporting), methodology (supporting), writing – review and editing (equal). **Antonio Rafael Quadros Gomes**: investigation (equal), formal analysis (supporting), methodology (supporting), visualization (supporting), writing – review and editing (equal). **Moises Hamoy**: resources (equal), supervision (equal), validation (equal), writing – review and editing (equal). **Eloisa Helena de Aguiar Andrade**: conceptualization (equal), project administration (lead), resources (lead), supervision (lead), validation (equal), writing – review and editing (equal).

## Funding

The article processing charge for the publication of this research was funded by the Coordenação de Aperfeiçoamento de Pessoal de Nível Superior—Brasil (CAPES – 001).

## Conflicts of Interest

The authors declare no conflicts of interest.

## Data Availability

The data that support the findings of this study are available from the corresponding author upon reasonable request.
